# Phenotypic Plasticity, Bet-Hedging, and Androgen Independence in Prostate Cancer: Role of Non-Genetic Heterogeneity

**DOI:** 10.3389/fonc.2018.00050

**Published:** 2018-03-06

**Authors:** Mohit Kumar Jolly, Prakash Kulkarni, Keith Weninger, John Orban, Herbert Levine

**Affiliations:** ^1^Center for Theoretical Biological Physics, Rice University, Houston, TX, United States; ^2^Institute for Bioscience and Biotechnology Research, University of Maryland, Rockville, MD, United States; ^3^Department of Physics, North Carolina State University, Raleigh, NC, United States; ^4^Department of Chemistry and Biochemistry, University of Maryland, College Park, College Park, United States; ^5^Department of Bioengineering, Rice University, Houston, TX, United States; ^6^Department of Physics and Astronomy, Rice University, Houston, TX, United States

**Keywords:** bet-hedging, stochasticity, androgen independence, non-genetic heterogeneity, phenotypic plasticity, intermittent androgen therapy

## Abstract

It is well known that genetic mutations can drive drug resistance and lead to tumor relapse. Here, we focus on alternate mechanisms—those without mutations, such as phenotypic plasticity and stochastic cell-to-cell variability that can also evade drug attacks by giving rise to drug-tolerant persisters. The phenomenon of persistence has been well-studied in bacteria and has also recently garnered attention in cancer. We draw a parallel between bacterial persistence and resistance against androgen deprivation therapy in prostate cancer (PCa), the primary standard care for metastatic disease. We illustrate how phenotypic plasticity and consequent mutation-independent or non-genetic heterogeneity possibly driven by protein conformational dynamics can stochastically give rise to androgen independence in PCa, and suggest that dynamic phenotypic plasticity should be considered in devising therapeutic dosing strategies designed to treat and manage PCa.

## Introduction

Phenotypic plasticity, the ability of cells/organisms in a population to switch states (phenotypes) in response to environmental conditions despite identical genetic contents, can have far-reaching consequences (1). In particular, it is widely acknowledged that the stochastic differentiation of a population of genetically identical cells (in other words, a clonal population) into distinct phenotypes can offer survival advantage in unpredictable fluctuating environments (2, 3). The phenomenon of bacterial persistence—the ability of a subpopulation of a clonal bacterial population to survive exposure to high concentrations of an antibiotic—is a striking example of the advantages of phenotypic plasticity (4). The existence of persisters protects the population from extinction under sudden harsh conditions and accounts for prolonged and recurrent infections (5). Recently, the concept of phenotypic plasticity has gathered much attention in cancer biology as well. Genetically identical cancer cells can manifest diverse phenotypes during tumor progression *via* mechanisms, such as epithelial–mesenchymal transition (EMT) (6), mesenchymal-amoeboid transition (6, 7), and neuroendocrine differentiation (8, 9). Such phenotypic plasticity can facilitate metastasis and therapeutic resistance in cancer cells (10, 11). These examples have illustrated the dire unmet need to investigate the underlying mechanisms regulating phenotypic plasticity and consequent non-genetic heterogeneity.

## Bacterial Persistence: A Hallmark of Phenotypic Plasticity

**Figure 1:**
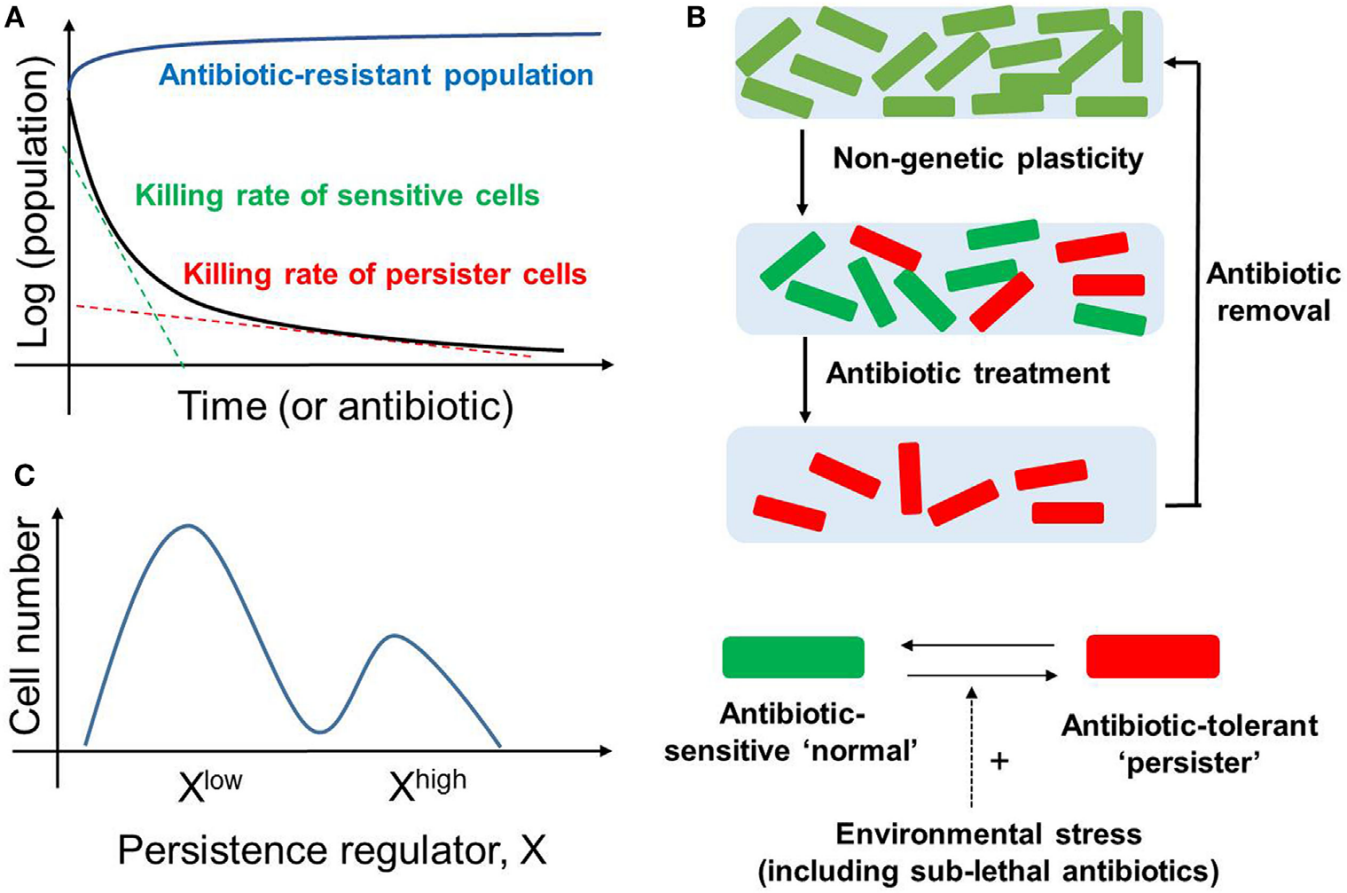
Bacterial Persistence. **(A)** Biphasic time-kill curve in bacterial populations exposed to antibiotics: faster killing rate of sensitive cell (green dotted line) followed by a slower killing rate (red dotted line) of persisters. In contrast, the antibiotic-resistant population continues to grow in presence of antibiotic (blue curve). **(B)** (top) An isogenic population of antibiotic sensitive cells can give rise to persisters *via* non-genetic/phenotypic plasticity. These slow-cycling persisters survive in the antibiotic treatment and tend to resume growth and generate a new population identical to the original population upon antibiotic removal (bottom). Persisters and non-persisters can switch among one another; the switching rate can be influenced by external stress factors. **(C)** Non-genetic heterogeneity of a key regulator of persistence (say X) in an isogenic population may give rise to two (or more) subpopulations that may continue switching stochastically among themselves to maintain persisters.

Many clonal bacterial populations respond to antibiotic drug treatment in a biphasic manner; the initial steep decrease in survival (fast killing rate) of a “normal” (drug-naïve) bacterial population is followed by a much slower decrease (slow killing rate), revealing the existence of persisters (4) (Figure 1A). These persisters, when isolated and regrown in the absence of drug, give rise to a population that is strikingly similar to the original population. When this population is exposed to the same antibiotic treatment, a similar time-kill curve is reproduced which was observed in the initial population, thereby indicating that the slower rate of killing of the persistent population is not permanent (Figure 1B). Thus, the phenomenon of persistence is different than that of resistance (defined as inherited ability of microorganisms, often due to genetic mutations, to grow at high concentrations of antibiotic irrespective of the duration of treatment) (4) (Figure 1A). Instead, bacterial persistence has been reported to act as a “phenotypic switch” where individual *E. coli* persisters stochastically transit into an actively growing state with their growth rate indistinguishable from the non-persisters and *vice-versa* (12) (Figures 1B,C). A lack of change in the persisters’ DNA sequence lends further credence to the idea that persistence is a non-genetic trait (13), i.e., the emergence of persisters need not depend on mutational or heritable changes in DNA sequence, but can result from diversity in cellular response to a repertoire of signals.

Direct single-cell and flow cytometry observations have suggested that persisters may arise as a subset of pre-existing dormant cells in an *E. coli* population (5). Specifically, some persister cells may have formed *a priori* even before the lethal antibiotic treatment. This pre-existing heterogeneity can be viewed as an example of “bet-hedging”—an evolutionary strategy that aims to maximize the fitness of an isogenic or a clonal population in dynamic environments through phenotypic heterogeneity, i.e., giving rise to two or more distinct subpopulations (14). Concomitant with this concept, bistability (existence of two distinct subpopulations that may reversibly transition to one another) in biochemical networks driving persistence has been proposed to give rise to persisters (15–17); this continued switching between different cell states can help to maintain a subpopulation of persisters (Figure 1C).

Another way of generating persisters is responsive diversification, where the application of sub-lethal levels of stress, including antibiotic treatment, can stimulate their formation (3, 5). Here, an initially homogeneous population can, while actively responding to the environmental change, generate stochastically different subpopulations of cells, *via* induced bistability in the underlying networks (18). The above-mentioned bacterial responses highlight how bacteria can deal efficiently with multiple antibiotics. Besides generating persisters, bacteria have been observed to display advanced social community skills, such as quorum sensing and developing biofilms to enhance their survival (19).

## Drug-Tolerant Persisters (DTPs) and Mutation-Independent Phenotypic Switching in Cancer

More complicated and complex counterparts of the social features discussed earlier often drive adaptive tumor dynamics (20–23), for instance, cooperation among cancer cells in evading chemotherapy (24) and in successfully colonizing distant organs (25–28). “Complicatedness” refers to the number and diversity of components in a tumor microenvironment (TME) (29)—besides widespread intratumor clonal heterogeneity (30), TME contains diverse cell types, such as endothelial cells, macrophages, fibroblasts, and other immune cells (31). On the other hand, “complexity” refers to the gamut of regulatory connections among those components (29)—tumor cells communicate among themselves and with these stromal cells *via* multiple mechanical and/or chemical cues, and can thus alter cellular phenotypes reversibly (32–40). For example, M1 and M2 macrophages can affect epithelial–mesenchymal plasticity oppositely (34), whereas mesenchymal breast cancer cells can polarize macrophages toward M2 polarization (35). Nonlinear dynamics emerging from this multi-scale crosstalk defines the adaptive evolution of tumors and can dictate therapeutic response (41, 42). Thus, with this combination of clonal diversity and non-mutational mechanisms, such as dynamic phenotypic plasticity, the tumor, as an ecosystem, can withstand many therapeutic assaults and present clinically insuperable challenges of tumor relapse, metastasis, and therapy resistance (19).

While the implications of clonal diversity leading to therapy resistance and devising effective therapeutic strategies have been well-appreciated (43, 44), contributions of cellular plasticity driven by intrinsic (for example, the hypoxic or metabolic state of a cell) and/or extrinsic (for example, the chemokines or matrix stiffness a cell is exposed to) signals—without any essential complicity of genetic mutations (45–49)—have only recently begun to be elucidated. Here, we focus on the striking parallels between bacterial persistence and resistance of prostate cancer (PCa) cells against androgen deprivation therapy (ADT). These parallels aim to better understand how cancer, a community of heterogeneous subpopulations (19), may benefit from bet-hedging and thus evade multiple, potent-targeted therapies, and appreciate how cancer can exhibit traits of a robust, diverse, and adaptive social ecosystem.

**Figure 2:**
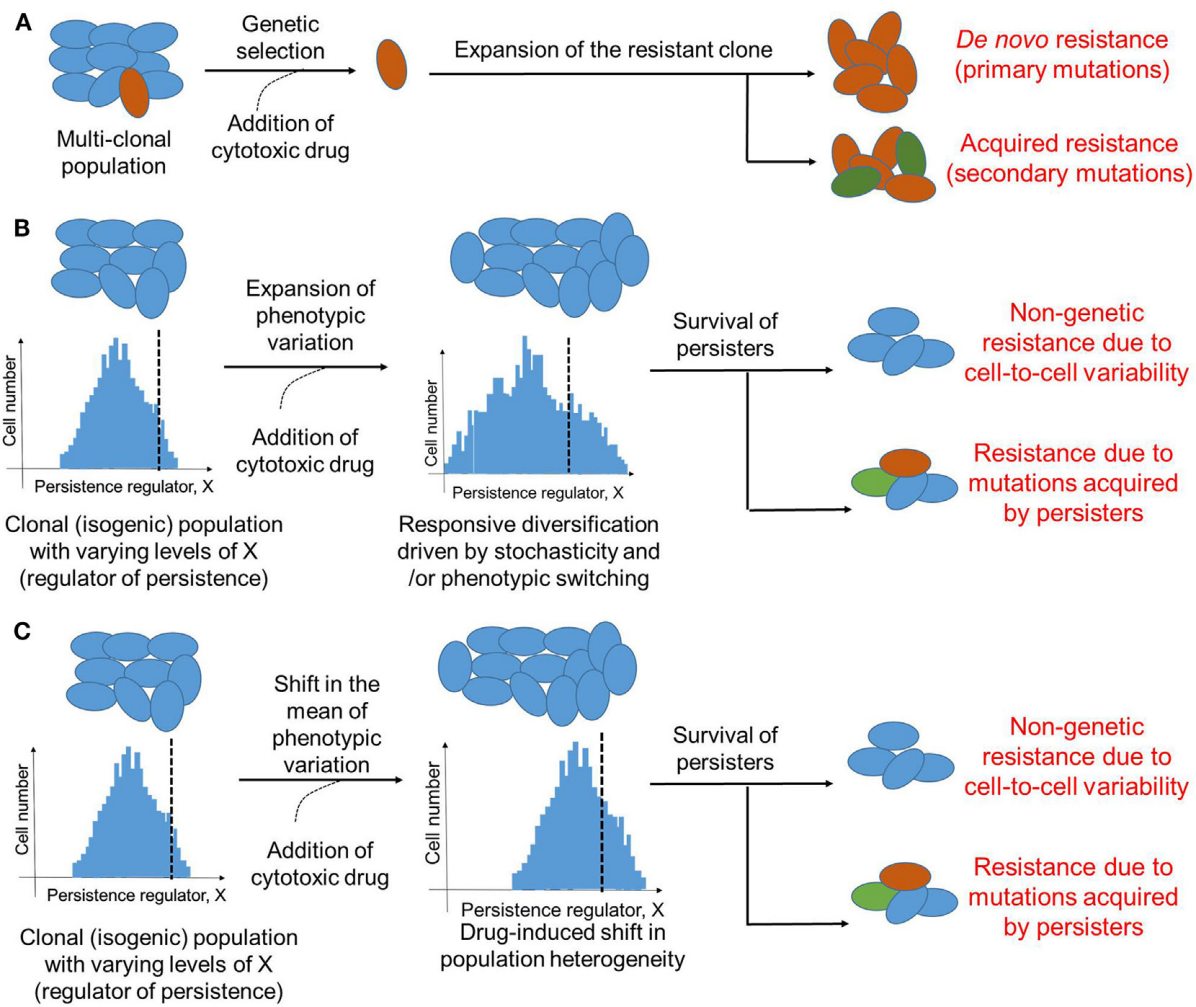
Modes of therapeutic resistance in cancer cells. **(A)** Cancer cells may resist cytotoxic drugs due to genetic mutations either pre-existing (*de novo*) in different clones that together constitute the cancer cell population, or those acquired during expansion of the drug-resistant clone. **(B)** An isogenic or a clonal population may be able to survive therapeutic assaults due to stochastic cell-to-cell variability of a key player regulating the formation of persisters. Drug treatment may enhance this non-genetic heterogeneity due to responsive diversification and/or drug-induced cellular reprogramming. This non-genetic heterogeneity can survive addition of cytotoxic drug, however, it can also lead to different acquired mutations by drug-tolerant persisters (DTPs) that can then be inherited by DTP clones. Dotted black line indicates which cells are persisters (to the right of it) versus which are not (to the left of it). **(C)** Same as **(B)**, but without any change in variation of the levels of X; instead, the mean levels of X change. Cells shown in one color represent identical genetic makeup.

Cancer has largely been considered a genetic disease driven by mutations (50). These primary and secondary mutations owing to clonal heterogeneity have been regarded as keystones of therapy resistance (51) (Figure 2A). However, the role of mutation-independent heterogeneity and phenotypic switching in cancer biology, such as cell-fate switching between a more dedifferentiated drug-resistant state and a well-differentiated drug-sensitive state in clonal or isogenic populations (32, 45), is gaining acceptance (46, 52). This dynamic cell-fate switching enables the emergence of multiple phenotypes from a single genotype, thus defying a precise linear genotype–phenotype mapping relationship and obfuscating the identification and targeting of mutations believed to be causal (53).

Striking recent observations in non-small cell lung cancer (NSCLC), melanoma, pancreatic ductal adenocarcinoma (PDAC), and breast cancer have illustrated the role of mutation-independent dynamic and adaptive phenotypic switching with implications in therapeutic design. For instance, treatment of multiple NSCLC cell lines sensitive to the epidermal growth factor receptor (EGFR) tyrosine kinase inhibition with a drug concentration 100-fold higher than the IC50 value led to the isolation of DTPs (45, 54). When propagated in drug-free media, DTPs resume growth and regain sensitivity to EGFR inhibition (45). This reversible phenomenon of persistence and the clonality of the population in which both persisters and non-persisters co-exist indicate that this phenotypic switching is mutation-independent (46). Similarly, some melanoma tumors that do not respond to B-raf proto-oncogene (BRAF) or mitogen-activated protein kinase inhibition may upregulate EGFR; this process can be reversed by discontinuing drug treatment, thereby re-sensitizing the apparently resistant cell population (54). Recent single-cell phenotyping and genome-wide transcriptomics reveal that in response to BRAF inhibition, many patient-derived BRAFV600-mutant cell lines undergo reversible cell-state transitions from a drug-naïve melanocytic state to a drug-resistant mesenchymal-like state (55). These transitions are driven not by selection of *de novo* genetically resistant clones, but instead result from the dynamics of underlying signaling networks (56) that can drive this adaptive transition (55). These instances of reversible and adaptive resistance against therapies are fundamentally different from *de novo* resistance (resistance due to “hard-wired” mechanisms, such as genetic mutations) and can help to explain clinical observations showing that some patients tend to regain sensitivity to BRAF inhibitor vemurafenib after a “drug holiday” (57). Furthermore, circulating tumor cells cultured from ER-positive/HER2-negative breast cancer patients revealed discrete HER2+ (proliferative) and HER2− (less proliferative, more drug-resistant) subpopulations that can interconvert spontaneously (58). Finally, a majority of PDAC cells were able to tolerate KRAS inhibition in both acute and sustained manner by adaptive switching through rewiring of signaling pathways (48). This switching did not invoke any significant mutational changes, underlining its non-genetic mechanism (48). These illustrative examples have motivated extensive investigations into phenotypic switching and DTPs in melanoma (47, 59, 60) and NSCLC (61), implying that drug resistance may be a reversible trait instead of a fixed modification, or that cells may dynamically enter and exit a window of drug resistance.

Similar mechanisms of phenotypic switching have been reported to regulate a dynamic equilibrium between cancer stem cells (CSCs)—a subpopulation with tumor-enhanced initiation potential and often enriched therapy resistance—and non-CSCs in breast cancer (62–64). These subpopulations have very similar, if not identical, genomic landscapes (62) and switching can be regulated by chromatin-mediated mechanisms (63), reminiscent of NSCLC studies (45), or cell–cell communication (32). Thus, similar to drug resistance, stemness need not be a static mutation-driven trait, but may be a functional reversible state that cancer cells can transiently adopt (46, 65, 66). Although the precise relation between DTPs and CSCs remains to be fully elucidated, mechanisms of drug resistance exhibited by CSCs and those by DTPs are remarkably similar (67).

## Role of Stochasticity and Cell-Cell Communication in Generating DTPs

Given that persistence tends to optimize the fitness of a clonal population by distributing the limited community resources into phenotypically distinct subpopulations (5), it is not surprising that cell–cell communication may be instrumental in generating DTPs and/or CSCs *via* bet-hedging and/or responsive diversification mechanisms. Similar to quorum sensing in bacterial persisters (5), cell–cell communication *via* soluble cytokines can maintain a dynamic equilibrium of CSCs and non-CSCs (32). Similarly, DTPs isolated from multiple breast cancer cell lines (68) display enhanced Notch-Jagged signaling (69), an evolutionarily conserved cell–cell communication pathway that can contribute to multiple hallmarks of cancer (70, 71), and potentially stabilize a persister cell state (72).

Further, similar to stress-induced dynamic responsiveness in bacteria, phenotypic transitions in cancer cells can be induced by therapy (68, 73, 74). One way these transitions could happen is by enhancing the pre-existing stochastic non-genetic heterogeneity (75–77) (Figure 2B); an alternative mechanism could be by altering the mean levels of a key regulator of cell survival (Figure 2C). Stochasticity is a fundamental feature of biological systems because all biochemical reactions may contain random fluctuations given that no two cells have the exact same number of key components, such as RNA polymerase, transcription factors, etc., that can affect gene expression or activity (78). Such cell-to-cell variability has been implicated not only in modulating the probability of differentiation of embryonic stem cells into varied developmental lineages (79, 80), but also in improving population survival by diversifying cells to be able to survive stressful conditions (81, 82), i.e., by “bet-hedging”. Stochastic single-cell behavior can also play a crucial role in recreating the population heterogeneity of breast cancer cells; apparently homogeneous subpopulations of breast cancer cells exhibiting distinct phenotypes, when cultured *in vitro* separately, often return to equilibrium populations over time (83). This inherent cell-to-cell variability can be enhanced by drug treatment by pushing a cell population to different cell states (55). Taken together, these observations argue for taking into account the inherent noise or stochasticity while assessing and optimizing anti-cancer therapies (84).

It should be noted that although DTPs are exemplars of non-genetic heterogeneity, genetic and non-genetic aspects of surviving therapeutic assaults may be intertwined. For instance, DNA damage—a key driver of genomic instability and genetic heterogeneity—can trigger persistence in *S. cerevisiae* by activating stress response (85). Similarly, induction of SOS response (response to DNA damage in which cell cycle gets arrested) increases the fraction of persisters in *E. coli* (86). On the other hand, EGFR T790M mutations were observed in NSCLC DTPs that were T790M-negative *a priori* (61, 87), indicating that DTPs provide a pool of cells from which various genetic modes of resistance can evolve (87) (Figure 2B). In the EGFR-addicted NSCLC cell line PC9 that initially revealed the existence of DTPs upon EGFR tyrosine kinase inhibition (45), 17 different persister-derived erlotinib-resistant colonies (PERCs) were established from a single persister (87). These PERCs displayed different genetic mechanisms of resistance, such as T90M mutation in EGFR and MET amplification (87). These two acquired resistance mechanisms account for over half of clinically reported cases that develop resistance against EGFR inhibitors (88). Furthermore, recent studies in melanoma, where vemurafenib treatment converted a transient transcriptional state in a clonal population into stable clones exhibiting resistance against vemurafenib (47) argue that genetic and non-genetic causes of resistance are not mutually exclusive. These observations are reminiscent of bacterial persisters acquiring stable resistance against antibiotics (89), and suggest that transient effects due to drug-induced cellular reprogramming and/or cell-to-cell heterogeneity may prevent cancer cells from extinction by giving them time to acquire inheritable secondary mutations that can stably drive the progression to relapse. Furthermore, given the growth-arrested state of persisters, the mechanism by which they gain mutation(s) may be independent of cell division, for instance, genome instability driven through DNA damage and consequent repair. Thus, preventing the formation of these persisters may contribute to reduced resistance.

## Non-Mutational Mechanisms of Androgen Independence in PCA

Prostate cancer is a leading cause of cancer incidence and cancer-related deaths in men. The 5-year survival rate of patients with local and regional PCa is almost 100%, but this rate drops to 28% in patients with metastasis to a distant organ (90). The primary standard of care therapy for locally advanced and metastatic PCa is ADT—surgical or chemical castration that lowers testosterone levels by stably suppressing androgen secretion (91, 92). This treatment has been in place for over 75 years, since Charles Huggins and colleagues described its efficacy in 1941 (92). While PCa patients typically respond well to ADT, most patients experience recurrence of the disease—termed as castration-resistant prostate cancer (CRPCa)—within 2–3 years of ADT (91). New treatments for CRPC, such as enzalutamide and abiretarone have been approved, but they extend median survival by merely 2–8 months (91), thus illustrating CRPC as an unmet urgent need.

Multiple mechanisms have been reported to contribute to resistance against ADT, such as increased expression of androgen receptor (AR), mutations in the ligand-binding domain of AR, and production of splice variants of AR (91) that can be upregulated in CRPC (93). Most frequently observed genetic aberrations in metastatic CRPC occur in AR, TP53, ETS family, RB1, and PTEN (94). Loss of PTEN function—often achieved by somatic mutations—has been correlated with worse survival (95) and can suppress the levels of androgen responsive genes by modulating AR activity (96). Loss of RB1 function enhances AR mRNA levels significantly and can induce resistance against ADT (97). Fusion of ETS family members, such as ERG to androgen-regulated gene TMPRSS2 can attenuate AR transcriptional activity, and thus drive selective pressure for development of PCa resistant to ADT (98). Also, inhibition of TP53 may diminish AR-mediated signaling (99). Thus, while no universal mechanism has been identified to drive evolution to CRPC, the AR pathway usually plays a key role (100, 101).

However, other non-mutational-based mechanisms similar to bacterial persistence may also contribute to this aggressive behavior. Metastatic CRPC has been reported to contain a mixture of cells displaying a range of AR expression levels (92). It is thus possible that this heterogeneity may exist *a priori* before the onset of ADT and/or is a product of responsive diversification, i.e., ADT induces the formation of these subpopulations from a clonal population. Recent evidence supports at least the former possibility, i.e., an isogenic population of PCa cells harbors a continuum of phenotypes with varying sensitivity to ADT, or, in other words, varying androgen-dependence. Different subclones established from a parental LNCaP cell line that is generally thought to be androgen-dependent had varying androgen sensitivity and AR activity levels that correlated with their different invasive and proliferative potential (102). Given that most of the differentially expressed genes among these clones were located on regions where no copy number variation was observed (102), the existence of these subclones possibly indicates a role of stochasticity or cell-to-cell variability in the control of AR activity levels.

Stochasticity or noise in a cell can arise due to multiple reasons. Besides the well-characterized transcriptional noise (103), there may be random fluctuations in the interaction networks themselves, especially those that comprise intrinsically disordered proteins (IDPs)—proteins that lack rigid 3D structures either along their entire length or in localized regions (104). Such promiscuity in interactions may give rise to “conformational noise” (104). IDPs have been found to be present as hub proteins in protein interaction networks from yeast to humans (105, 106), thus significantly impacting biological information transfer and propagating noise in signaling pathways. In contrast to well-defined energy landscapes of ordered proteins that determine their structure, IDPs may dynamically populate an ensemble of interconvertible structural conformations due to many local energy minima separated with low-energy barriers (107), especially when overexpressed (108). Several well-known oncogenes and tumor suppressor proteins, such as p53 (109), BRCA1 (110), PTEN (111), c-MYC (112, 113), and KRAS (114), and other key players regulating the formation of CSCs, such as LIN28, OCT4, NANOG, and SOX2 (115) have been reported to contain intrinsically disordered regions (IDRs). Further, many core modulators of EMT—a mechanism of phenotypic plasticity that shares molecular and functional overlaps with CSCs (116)—was predicted to contain IDRs (117). In the context of PCa, a striking example of an IDP is the key target of ADT itself, AR (118). Similarly, a majority of cancer/testis antigens (CTA)—a heterogeneous group of proteins that are typically expressed in testis with little or no expression in most somatic tissues, but aberrantly expressed in PCa—have been reported as IDPs (119).

Intrinsically disordered proteins may undergo a disorder-to-order transition to varying extents upon interacting with a cognate ligand, or upon specific post-translational modifications prior to ligand interaction (113, 120–122). Moreover, IDPs tend to have faster kinetics of interaction with their partners (faster binding/unbinding rates) (123), potentially amplifying promiscuity in interactions, and increasing stochasticity by allowing more flexibility in conformational switching. Considered together, these observations underscore the role of IDPs/IDPRs in phenotypic switching and thus the adaptability of biological systems in hostile environments (124, 125).

Our recent work employing multiple biophysical approaches illustrated how intrinsic disorder in a CTA named prostate-associated gene 4 (PAGE4) (126) can lead to its different conformations with implications for response to ADT (127). PAGE4 is a stress-response protein that is upregulated in response to many stress factors, such as inflammation; it is undetectable in normal adult glands, but aberrantly expressed in diseased gland and in prostatic lesions infiltrated with inflammatory cells (128). Epithelial PAGE4 correlates with and is an independent predictor of survival for patients with hormone-naïve PCa (129). PAGE4 is associated with attenuated AR signaling (129); one of the underlying mechanisms appears to involve the ability of PAGE4 to potentiate the transcription factor activator protein-1 (AP-1) (130) that can negatively regulate AR activity (131, 132). PAGE4 is phosphorylated by another component of the stress-response pathway homeodomain-interacting protein kinase 1 (HIPK1) predominantly at T51 which is critical for its ability to potentiate the transactivation of c-Jun (133), the most potent transcriptional activator of the AP-1 complex (134). PAGE4 is hyper-phosphorylated by CDC-like kinase 2 (CLK2) at many S/T residues, including T51. The interaction of PAGE4 with these two kinases leads to opposite functions. HIPK1-phosphorylated PAGE4 (HIPK1-PAGE4) potentiates c-Jun, while CLK2-phosphorylated PAGE4 (CLK2-PAGE4) attenuates c-Jun activity. This functional difference most likely arises from the different conformations of the PAGE4 ensemble, as elucidated using small-angle X-ray scattering, single-molecule fluorescence resonance energy transfer, and multidimensional NMR. HIPK1-PAGE4 exhibits a relatively compact conformational ensemble that binds AP-1, but CLK2-PAGE4 is more expanded and attains a random-coil conformation with less affinity for AP-1 (127).

**Figure 3:**
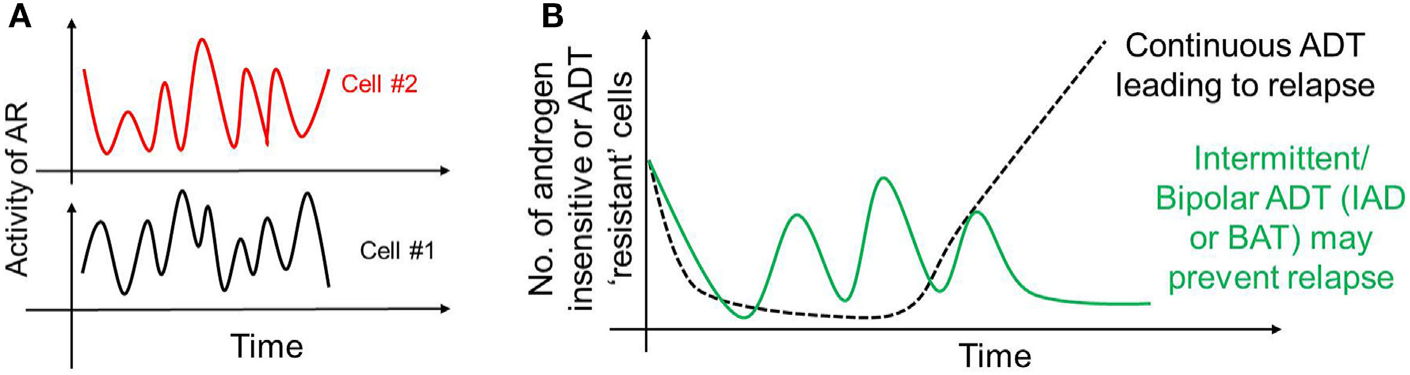
Non-genetic heterogeneity in prostate cancer. **(A)** Androgen receptor (AR)/prostate-associated gene 4 (PAGE4)/activator protein-1 (AP-1) circuit can give rise to oscillations of AR activity in a cell that can dynamically vary its dependence on androgen. These oscillations need not be synchronized across the population. **(B)** These oscillations, together with any other mechanisms of persistence, may survive a continuous androgen deprivation therapy and eventually regrow the entire population leading to tumor relapse (dotted black curve). However, “drug holidays,” such as intermittent androgen deprivation or bipolar androgen therapy may convert persisters to drug-sensitive cells, thus always keeping the number of androgen-independent (resistant) cells in check (solid green curve).

As mentioned above, AP-1 can inhibit AR activity; moreover, AR can transcriptionally inhibit CLK2 (127), thereby forming a negative feedback loop in PAGE4/AR/AP-1 interactions. A recent mathematical model has predicted that this feedback loop can give rise to sustained or damped oscillations in the levels of AR activity, HIPK1-PAGE4 and CLK2-PAGE4 (Figure 3A), suggesting that androgen dependence of a cell can be a dynamic trait. Therefore, as the intracellular levels of HIPK1-PAGE4 and CLK2-PAGE4 vary dynamically, cells can go on phenotypic excursions with varying insensitivities to ADT [cells “resistant” to ADT have typically increased AR activity as an adaptive auto-regulatory mechanism (135)]. Additional interactions of these components could convert these oscillations into a multistable system. As already emphasized above, this heterogeneous population can thus potentially better evade the effects of ADT as compared to a homogeneous PCa population. This non-genetic mechanism is in contrast with the Darwinian clonal evolution model (136) which assumes the existence of mutually exclusive androgen-dependent and androgen-independent clones. Thus, in addition to genetic changes, phenotypic plasticity in PCa may be driven by underlying dynamics of the PAGE4/AP-1/AR circuit.

Another plasticity mechanism that has been recently reported to be associated with PCa relapsing from antiandrogen therapies is where PCa cells acquire morphologic features of neuroendocrine carcinoma, a cell lineage whose survival no longer depends on AR (137, 138). Similar to the observed plasticity between epithelial and mesenchymal phenotypes in breast cancer (6, 52), between proneural and mesenchymal phenotypes in glioma (139), and between neuroendocrine and mesenchymal phenotypes in small cell lung cancer (9), this more macroscopic plasticity in PCa may mediate cellular response to multiple therapies (8, 140) and serve as a hallmark for aggressive disease progression (141).

## Implications of Dynamic Phenotypic Plasticity and Stochastic State Switching in Therapeutic Design

Resistance against various therapies can unquestionably result from secondary mutations (142–145) and/or pre-existing clones with specific genetic changes (43, 146–149). But, non-mutational stochastic cell-to-cell variability that can affect drug response and therapy-induced cellular reprogramming can also drive acquired resistance (45, 46, 52, 60, 61, 68, 73, 87, 150–152). Thus, similar to precision medicine attempts focusing on genomic landscape differences (153), effective therapeutic dosing strategies, and target identification calls for considering the effects of non-genetic heterogeneity and therapy-induced phenotypic plasticity that may give rise to persisters.

The existence of these persisters may offer a plausible explanation for the success of interval dosing therapeutic strategies in stalling tumor growth in many cancer types (57, 154–156). Such discontinuous treatment regimens may exploit the fitness disadvantage typically exhibited by the DTPs in the absence of drug (157), thereby leading to a regression of persister subpopulation. Particularly, in the context of PCa, treatment paradigms that involve cycles of ADT followed by no ADT [referred to as intermittent androgen deprivation (IAD)] (158) or ADT followed by supra-physiological dose of androgen [referred to as Bipolar ADT or bipolar androgen therapy (BAT)] (159) may be as good in terms of disease-free survival rates. Continuous ADT can result in a sustained pool of PCa persisters that may provide a latent reservoir of cells that can eventually acquire diverse genetic mutations accounting for stable drug-resistance, while intermittent ADT may discourage the maintenance of persisters, thus restricting phenotypic heterogeneity and resulting in higher disease-free survival rates (Figure 3B). Thus, an intermittent approach is likely to be more potent in targeting the vulnerabilities of different subpopulations at once, as compared to a continuous therapy treatment that can not only spare a set of recalcitrant population, but also stabilize a transient mechanism of drug resistance (160).

An alternative approach to intermittent or discontinuous dosing strategy is combinatorial therapy. A recent study that analyzed both human clinical trial data and the drug responses of various patient-derived xenografts (PDXs) highlighted how combinatorial therapy can be beneficial even without any synergy in drug actions, due to patient-to-patient variability (161). Combinatorial therapy has also shown initial promise in PTEN-deficient PCa, where PI3K and AR signaling inhibit each other, potentially generating multiple subpopulations (162). Inhibiting either pathway singly activates the other, enabling adaptive response. However, pharmacological inhibition of both these pathways causes almost complete regression of the disease both in PTEN-deficient PCa mouse models and in human prostate PDXs (162).

Combinatorial therapy can also help to target the vulnerabilities of DTPs. Goldman et al. (68) observed that the treatment of breast cancer cells with high concentrations of taxanes generates persisters that drive aggressive tumor formation *in vivo*. These persisters display activated Src family kinase/hemopoietic cell kinase pathways whose pharmacological inhibition in a temporally constrained manner led to enhanced apoptosis (68). Similarly, Deb et al. (163) identified two mutually exclusive clonal subpopulations in altered signaling states—one with upregulated pSTAT3 and the other with downregulated SMAD2/3—and targeted STAT3 and BCL6 (a transcription factor downstream of SMAD2/3) in a combinatorial manner to overcome non-genetic heterogeneity. Furthermore, dual inhibition of Wnt and Yes-associated protein (YAP) signaling can restrict the population of both epithelial-like and mesenchymal-like CSCs (164). These combinatorial therapies are reminiscent of combinations of drug pyrazinamide (that specifically targets *M. tuberculosis* persisters) with other canonical treatments (165). However, persisters in both bacterial and cancer cell populations can often be heterogeneous in their mechanisms and extent of drug-tolerance (4, 47, 150, 166). In fact, the very idea of defining IC_50_ (50% inhibitory concentration—the drug concentration where the viability of the population is half as that of the control case) implies that individual cells in a given population exhibit heterogeneous response to treat the cells with drug concentrations considered to be lethal (165). Valuable insights into the extent of heterogeneity can be gauged by other pharmacological parameters, such as the variability in maximum susceptibility of all cells in a given population to cell death, and the range of doses over which different subpopulations get killed (165).

Given that ADT has remained the primary standard of treatment for advanced/metastatic PCa for more than 75 years, we envisage that the conceptual framework outlined above can help to guide alternative treatment options. For example, clinicians may consider prescribing IAD or BAT, thus sparing the patient of the huge costs and undesirable side effects of chronic androgen deprivation. Indeed, in a recent report, albeit on just three cases, non-metastatic PCa patients were treated effectively with long-term primary IAD (167). Although IAD is not a standard therapy for patients with non-metastatic PCa, this exploratory clinical study underscores the benefits of challenging the cancer cell’s adaptive robustness due to its innate phenotypic plasticity. While the debate over the merits and controversies of administering either ADT regimen continues, and convincing data are needed to favor one over the other (157, 168), we trust the arguments presented here may inspire clinicians to reconsider treatment options and management of PCa that is currently estimated to strike one in every six men in the USA.

Phenotypic plasticity need not be a mechanism specific to PCa—it may also help normal prostate cells to cope with the significant diurnal variation (20–25%) in circulating testosterone levels in men (169). Thus, it is possible that as an adaptive evolutionary mechanism, PCa cells may be highly adept in phenotypic plasticity and persisting as a response to chronic and high fluctuations in hormonal levels and aggressive ADT—both of which represent the frequency of stressful conditions that can tune the rates of switching, and hence the frequency of persisters (170).

## Conclusion

Phenotypic plasticity allows for a clone to sample many phenotypes—each with varying sensitivities—thus generating mutation-independent heterogeneity and enhancing clone survival. Therefore, phenotypic plasticity may serve as an effective bet-hedging strategy that may help overcome the varying selection pressures faced by a tumor (171). Here, we argue that besides genetically encoded resistance to ADT, PCa recurrence may also emerge from a phenomenon that bears a close resemblance to bacterial persistence—a bet-hedging strategy to face unpredictable harsh environmental fluctuations by generating non-genetic or mutation-independent phenotypic heterogeneity. Two crucial mechanisms underlying this heterogeneity—stochastic cell-to-cell variability and drug-induced cellular reprogramming—have already been implicated in forming DTPs. Here, we present one potential implementation strategy for generating cell-to-cell variability—the protein conformational dynamics of an intrinsically disordered protein PAGE4—that can generate dynamically varying AR levels in a cell, and thus give rise to different subpopulations, each with a varied sensitivity toward ADT. This “bet-hedging” may facilitate the presence of persisters—drug-tolerant reservoirs of cells from which multiple mechanisms of drug-resistance may evolve. Modulating inherent dynamic phenotypic plasticity and consequent heterogeneity may increase therapeutic efficacy.

## Author Contributions

MKJ, PK, and HL conceptualized the idea. MKJ and PK wrote the first draft. JO and KW contributed to revising the manuscript.

## Conflict of Interest Statement

The authors declare that the research was conducted in the absence of any commercial or financial relationships that could be construed as a potential conflict of interest. The reviewer ML and handling editor declared their shared affiliation.
